# Supplementary figures and images for: Reference Values of Cerebral Artery Diameters of the Anterior Circulation by Digital Subtraction Angiography: A Retrospective Study
Source: Diagnostics (Basel). 2022 Oct 12;12(10):2471. doi: 10.3390/diagnostics12102471 (PMC9600370; doi:10.3390/diagnostics12102471)

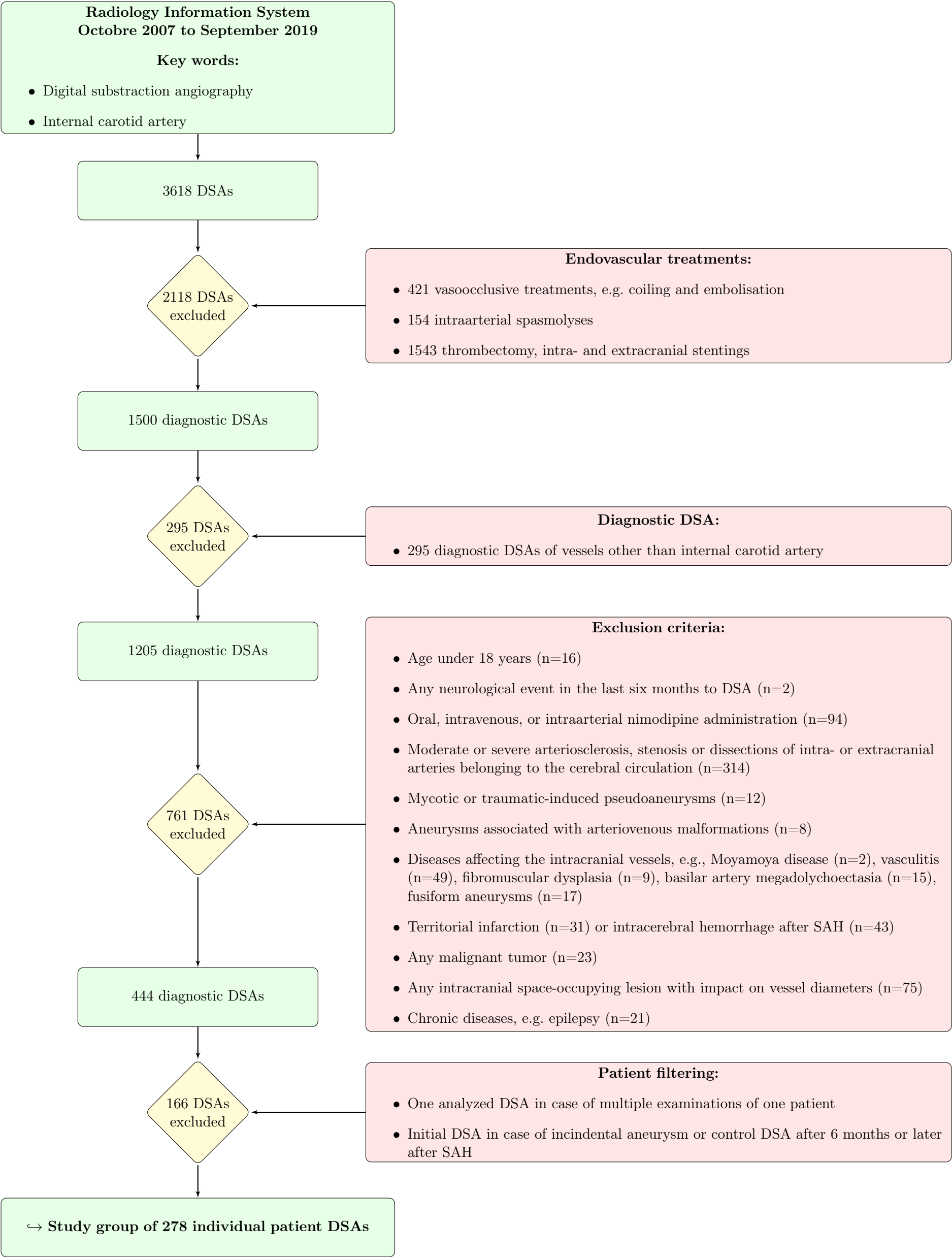

Supplement: Supplementary file 1 [file diagnostics-12-02471-s001.zip › Figure_S1.pdf]
